# Subspace Complexity Reduction in Direction-of-Arrival Estimation via the RASA Algorithm

**DOI:** 10.3390/s25196120

**Published:** 2025-10-03

**Authors:** Belan Bapir-Bakr, Haitham Kareem-Ali, Sandra Gutiérrez-Serrano, Nerea del-Rey-Maestre, Carlos Hernández-Fernández

**Affiliations:** 1 Signal Theory and Communications Department, Superior Polytechnic School, University of Alcalá, Campus Universitario, 28805 Alcalá de Henares, Madrid, Spain; belan.bapir@edu.uah.es (B.B.-B.); sandra.gutierrezs@uah.es (S.G.-S.); c.hernandezf@edu.uah.es (C.H.-F.); 2 Computer Engineering Department, Tishk International University, Kurdistan Region, Iraq; 3 Department of Communication Engineering, Sulaimani Polytechnic University, Salaimani 46001, Iraq; haitham.ali@spu.edu.iq

**Keywords:** DoA estimation, correlation, subspace, projection matrix construction, sampling methodology

## Abstract

The complexity and scale of contemporary datasets are increasing, making the need for reliable and effective subspace processing more pressing. In array signal processing, the quality of the projection matrix and the structure of the noise subspace have a significant impact on the Direction of Arrival (DoA) estimation accuracy. In this study, the limits of typical subspace sampling approaches are emphasized, especially when source coherence, restricted snapshots, or low Signal-to-Noise Ratio (SNR) are present. Traditional DoA estimate strategies are revisited. To overcome these problems, a selective subspace refinement-based enhanced dimensionality reduction technique is proposed. Using a correlation measure based on the $\ell_{2}$-norm, the suggested strategy minimizes the projection subspace by finding and keeping just the noise subspace’s least correlated columns. Adaptively choosing the first, last, and least dependent inner eigenvectors allows the method to maintain excellent angular resolution and estimation accuracy while drastically reducing computational complexity by up to 75%. This correlation-aware subspace design enhances the final pseudo-spectrum’s robustness, numerical stability, and orthogonality. The suggested method provides a scalable and effective solution for high-resolution DoA estimation in data-intensive signal environments, as demonstrated by experimental results that show it beats traditional methods in terms of accuracy and execution time.

## 1. Introduction

Over the years, sensor array source localization based on DoA estimation has drawn interest from a variety of engineering fields [1], such as sonar systems [2], emergency-call localization [3], automatic vehicle tracking [4], radar systems [5], and patient monitoring [6]. To obtain DoA information for these applications, the impinging signals on the antenna array are usually digitally processed using a specific direction-finding technique [7]. Furthermore, the performance of Multiple Input Multiple Output (MIMO) systems is improved by the implementation of adaptive beamforming and efficient direction-finding methods [8], in the development of smart antenna technology, which places nulls in the direction of interfering sources to suppress interference [9]. Recently, reconfigurable intelligent surfaces (RISs) have emerged as low-power, programmable metasurfaces that shape propagation to enhance coverage, suppress interference, and improve localization/DoA accuracy [10]. Associated developments in harmonic coordinated beamforming for automobile radar enhance lane detection and DoA-based sensing [11]. The space-time path [12], which is required for MIMO systems to operate efficiently [13], can be precisely represented using a source localization technique. Improving direction-finding performance in huge MIMO systems requires efficient sampling of the received data matrix. Therefore, the difficult problem of producing a smaller dataset closely reflective of a bigger dataset may be solved using extreme value theory, which is also very practical [14].

The literature has presented a number of algorithms and approaches related to the DoA estimation problem, such as Minimum Variance Distortionless Response (MVDR) [15], Multiple Signal Classification (MUSIC) [16], Minimum Norm (MinNorm) [17], and Estimation of Signal Parameters via the Rotational Invariance Technique (ESPRIT) [18]. In MinNorm, the optimal array weight vector is used to reduce the output norm. Only Uniform Liner Array (ULA) configurations are appropriate for this algorithm. In Root-MUSIC, polynomial root searching replaces spectral searching in order to simplify classical music [19]. While efficient, this method is sensitive to coherent sources and limited to ULA geometry. The computationally inefficient MVDR method requires the inverse of the Covariance Matrix (CM). In contrast, other techniques require eigenvalue or singular value decomposition of the data matrix to determine eigenvalues and eigenvectors [20]. Additionally, eigen/singular value decomposition methods are inefficient in terms of processing complexity [21]. It is challenging to distinguish between noise and signal subspaces, especially in poor channel circumstances [22].

Alternatively, effective linear DoA estimating algorithms avoid CM factorization difficulty. The algorithms Propagator [23] and Orthogonal Propagator [24] divide the CM into two smaller matrices. These strategies, suggested as an alternative to the traditional MUSIC algorithm, reduced complexity significantly. Also, reconfigurable full-digital, phase-interferometric FPGA AoA has demonstrated real-time RTLS with tunable phase resolution [25].

However, processing the entire CM is not practical in a large MIMO system. Numerous methods have been presented based on Propagators’ notion [24,26]. The methods are computationally efficient and do not need CM building. Nevertheless, these methods typically require a large array aperture to achieve satisfactory performance.

In Column Subset Selection (CSS), a subset of columns is extracted from a high-dimensional matrix so as to create a column submatrix that is an accurate representation of the original matrix [27]. As a result of this method, the complexity of signal processing is significantly reduced [28]. In recent years, the concept of sampling the CM has been proposed as a method for constructing the projection matrix (PM) from this perspective [29,30]. The influence of the quantity of sampled columns from the CM on the formulation of the PM has been investigated in [28]. The DoA of incident signals can be accurately estimated by selecting L-columns from the CM, where L represents the number of sources. Moreover, it has been demonstrated that increasing the number of sampled columns enhances DoA estimation accuracy and improves the Degree of Freedom (DoF), albeit at the cost of increased computational complexity in PM construction. Nevertheless, the authors are unaware of any optimal criterion for determining which columns of the CM should be utilized for PM construction in the literature. Typically, traditional methods use the CM’s first L columns as the default Classical sampling (CS) technique [31,32,33].

This methodology is poor because the chosen columns are predetermined (i.e., the first L columns) and fail to sufficiently capture the informative richness of the original CM. To overcome this limitation, Uniform Sampling (US) was proposed in [29], which increases DOF without increasing extracted columns to improve DoA estimation performance. As compared to the conventional technique, the US distribution increases DOF from (C−1) to (CU+U−1), where C is the number of sampled columns and U is the ratio between the number of array elements and the number of sources. A new approach, Non-Uniform Sampling (NUS), improves DoA estimation accuracy by removing energy-rich columns [30]. The NUS principle uses random column selection in the CM to create a sampling matrix. Applying the NUS approach results in higher energy retention for signal eigenvalues, resulting in a higher number of DoAs detected compared to the traditional technique.

Apart from sampling strategies, another important challenge in DoA estimation is the presence of signal correlation, particularly in environments with multipath or coherent sources. To mitigate this, the Spatial Smoothing (SS) technique is commonly applied, which divides the array into overlapping subarrays [34]. While effective in decorrelating signals, SS may reduce resolution and increase computational cost. These constraints require more complicated and permanent solutions. To address these limitations, Enhanced Spatial Smoothing (ESS) was proposed in [31], enabling more accurate DoA estimation for coherent sources [35]. To further address the limitations of earlier ESS techniques, the ESS-SS variant was proposed, which applies ESS directly to the smoothed covariance matrix to solve subarray information underutilization in previous ESS techniques [36]. This approach lowers noise and preserves subspace structure. However, subarray averaging and multiple covariance reconstructions become computationally expensive. To mitigate this complexity, [37] proposed a real-valued MUSIC method that avoids complex eigen-decomposition and reduces complexity by about 75%. It keeps accuracy near that of traditional MUSIC and supports arbitrary array configurations. Its resolution may be limited under low SNR or coherent signal situations, though, because it ignores subspace redundancy and inter-column correlation.

In addition to these traditional methods, machine learning (ML) techniques have been investigated recently for estimating DoA. Several examples include CNN-assisted estimators for robust Capon processing [38], multi-task CNN frameworks that jointly address DOA and noise power estimation [39], and DeepMUSIC [40], which uses deep neural networks to improve the classical MUSIC spectrum. Although promising, these techniques usually necessitate large labeled training datasets and may encounter domain mismatch when used in various propagation conditions or array geometries.

DoA estimation is still difficult due to a number of real-world issues, such as low SNR, few snapshots, array size restrictions, and source correlation, even with the success of Standard MUSIC and its variations. Current approaches frequently compromise computational complexity for resolution or exhibit poor generalization across array configurations. Nevertheless, during the subspace reduction process, the inter-column correlation structure inside the noise subspace has not been explicitly taken into consideration by any of the previously described sampling techniques. A high correlation between noise eigenvectors is known to adversely affect the formulation of the PM, which reduces the effectiveness of DoA estimation methods. A correlation-aware subspace refinement approach is used to solve this problem, in which columns from the noise subspace $Q_{n}\in C^{M\times (M-L)}$ are adaptively chosen according to their pairwise decorrelation efficiency. The goal is to reduce redundancy among the chosen noise eigenvectors in order to improve DoA estimation resolution and accuracy. This is accomplished by calculating the noise subspace’s correlation matrix and assessing each eigenvector’s correlation norm, each of which measures the overall correlation between a particular column $C_{i}$ and every other column. A lower norm value is preferred for subspace building, since it shows less mutual dependency. The first column, the last column, and the inside column with the lowest correlation norm constitute the selection procedure. In the condensed subspace, these choices ensure low redundancy, edge representation, and structural variety. In addition to being robust and information-rich, the resulting projection matrix is numerically stable, providing enhanced resolution and noise tolerance, particularly in difficult situations like source coherence, low SNR, or limited snapshots.

The following is a summary of this paper’s primary contributions:

- RASA, a structured LCCS-based algorithm that consistently chooses minimum correlated vectors to minimize the dimensionality of the noise subspace. To accomplish this, a correlation-aware sampling technique is used, which evaluates column-wise $\ell_{2}$-norms to find low-redundancy directions and computes Pearson correlation coefficients from the real half of the noise subspace. The resultant smaller subspace greatly reduces processing cost while maintaining excellent resolution and estimation accuracy.
- A generic steering vector formulation is developed to ensure compatibility with arbitrary array geometries, including ULAs, URAs, and their non-uniform counterparts (NULA and NURA). While all main simulations are conducted on the ULA configuration, additional results with NULA and NURA confirm that the RASA retains effectiveness under irregular sensor placements.
- To assess the algorithm’s performance under various SNR levels, snapshot counts, and array topologies, a thorough simulation study is carried out.
- Through a correlation-aware sampling technique to select and keep minimally correlated columns from the noise subspace, RASA decreases computational cost, improves orthogonality, decorrelation, and estimate accuracy under difficult signal conditions.

The rest of this paper is structured as follows: Section 2 presents the DoA estimation model using arbitrary array geometry, while Section 3 covers PM construction principles, including sampling methodologies for PM. Section 4 introduces the proposed Reduced Angle Subspace Algorithm (RASA), outlining its technique and theoretical assessment. In Section 5, comparisons of DoA algorithms are shown and analyzed. Section 6 presents the simulation results. In Section 7, we summarize our paper and conclude our findings.

## 2. An Arbitrary Array-Based DoA Estimation Model

**Figure 1:**
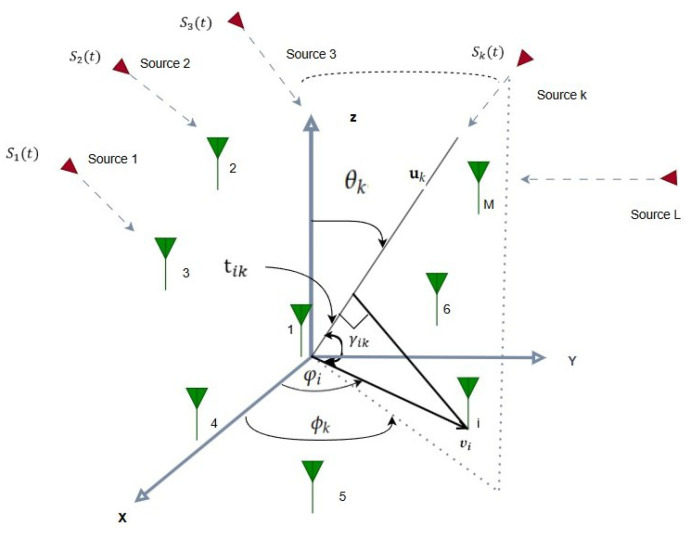
DoA model with M-arbitrary elements and L arriving signals.

A receiving antenna array composed of *M* omnidirectional elements collects narrowband signals transmitted by *L* far-field sources, as illustrated in Figure 1.

The signals that impact the array randomly are represented by ${\left(\theta_{l},\phi_{l}\right)}_{l=1,2,...,L}$ where $\theta_{l}$ and $\phi_{l}$ represent the elevation and azimuth angles of the ith signal, respectively. To optimize the subsequent procedures, the array outputs are initially down-converted to baseband and subsequently processed digitally to estimate the DoA. The comprehensive received signal vector, encompassing the contributions from incoming sources and additive Gaussian noise, is represented as follows:

(1) x(t)=A(θ,ϕ)s(t)+n(t)

Assuming $s\left(t\right)={\left[\begin{array}{cc} s_{1}\left(t\right), & s_{2}\left(t\right),\ldots ,s_{L}\left(t\right) \end{array}\right]}^{T}\in C^{L\times 1}$ is a modulated signal vector, $n\left(t\right)={\left[\begin{array}{cc} n_{1}\left(t\right), & n_{2}\left(t\right),\ldots ,n_{M}\left(t\right) \end{array}\right]}^{T}\in C^{M\times 1}$ represents the additive white Gaussian noise associated with each channel, and each steering vector $a\left(\theta_{k},\phi_{k}\right)\in C^{M\times 1}$ denotes the spatial response of the array to the *k*th incident signal arriving from the direction $\left(\theta_{k},\varphi_{k}\right)$. The vectors collectively constitute the array manifold matrix $A\left(\theta ,\varphi \right)\in C^{MxL}$, encompassing the steering vectors associated with *L* incoming sources. This matrix is delineated as follows:

(2) $$A\left(t\right)=\left[\begin{array}{cc} a(\theta_{1},\phi_{1}), & a\left(\theta_{2},\phi_{2}\right),\ldots ,a\left(\theta_{L},\phi_{L}\right) \end{array}\right]\in C^{M\times L}$$

To adopt the arbitrary array configuration shown in Figure 1, we need to drive the steering vector for the *k*th plane wave incident on this array. i.e., $a(\theta_{k},\phi_{k})$. A unit vector $u_{k}$ containing both $\theta_{k}$ and $\phi_{k}$ can be defined according to the established spatial modeling protocols in 3GPP TR 38.901 [41] as Equation (3). Here, the unit vectors for Cartesian co-ordinates are described by ${\hat{a}}_{x}$, ${\hat{a}}_{y}$, and ${\hat{a}}_{z}$.

(3) $$u_{k}=\cos\phi_{k}\sin\theta_{k}{\hat{a}}_{x}+\sin\phi_{k}\sin\theta_{k}{\hat{a}}_{y}+\cos\theta_{k}{\hat{a}}_{z}$$

Next, we must establish a new unit vector $\upsilon_{i}$ as is expressed in (4) in order to compute the distance between the positions of other elements and the reference element (i.e., element 1, see Figure 1):

(4) $$\upsilon_{i}=r_{i}\cos\varphi_{i}{\hat{a}}_{x}+r_{i}\sin\varphi_{i}{\hat{a}}_{y}+z_{i}{\hat{a}}_{z},\;i=1,2,\ldots ,M$$

Here, $r_{i}$ denotes the radial distance from the *z*-axis; $\varphi_{i}$ represents the azimuthal angle in the *x*–*y* plane; $z_{i}$ indicates the vertical height of the *i*-th sensor element; and ${\hat{a}}_{x}$, ${\hat{a}}_{y}$, ${\hat{a}}_{z}$ are the unit vectors aligned with the *x*, *y*, and *z* axes, respectively. The angles between the unit vectors for the *i*-th sensor with respect to the reference element $u_{k}$ and $\upsilon_{i}$ can then be computed as follows, where the Euclidean norm is shown by ∥·∥:

(5) $$\gamma_{ik}=\cos^{-1}\left(\frac{\upsilon_{i}\cdot u_{k}}{∥\upsilon_{i}∥∥u_{k}∥}\right)$$

The entire set of $\gamma_{ik}$, which represents the angular relationships between *M* elements and *L* sources, is shown in Equation (6) and is represented by the matrix $\Gamma_{ik}$.

(6) $$\Gamma_{ik}=\left(\begin{array}{ccccc} \gamma_{11} & \ldots & \gamma_{1k} & \ldots & \gamma_{1L}\\ \gamma_{21} & \ldots & \gamma_{2k} & \ldots & \gamma_{2L}\\ \vdots & \ddots & \vdots & \ddots & \vdots \\ \gamma_{M1} & \ldots & \gamma_{Mk} & \ldots & \gamma_{ML} \end{array}\right)$$

The difference in distance is used to calculate the relevant time delay associated with each $\gamma_{ik}$ in the following manner:

(7) $$t_{ik}=r_{i}\cos\gamma_{ik}=r_{i}\left\{\sin\theta_{k}\cos\left(\phi_{k}-\varphi_{i}\right)+z_{i}\cos\theta_{k}\right\}$$

Likewise, the entire set of $t_{ik}$ can be represented as follows using an M×L matrix:

(8) $$T_{ik}=\left(\begin{array}{ccccc} t_{11} & \ldots & t_{1k} & \ldots & t_{1L}\\ t_{21} & \ldots & t_{2k} & \ldots & t_{2L}\\ \vdots & \ddots & \vdots & \ddots & \vdots \\ t_{M1} & \ldots & t_{Mk} & \ldots & t_{ML} \end{array}\right)$$

Finally, the angular phase difference $\omega_{ik}$ can be calculated by multiplying $t_{ik}$ and the propagation constant β as is expressed in Equation (9), where λ is the signal wavelength.

(9) $$w_{ik}=\beta t_{ik}=\frac{2\pi }{\lambda }t_{ik}$$

The total $w_{ik}$ that correlate to $T_{ik}$ are shown as follows, just like in Equations (6) and (8). In this case, the *M* sensor elements are represented by the row index i=1,…,M, while the *L* arriving sources are represented by the column index k=1,…,L. The angular phase difference between the *i*-th sensor and the *k*-th source is represented by each entry $\omega_{ik}$:

(10) $$\omega_{ik}=\left(\begin{array}{cccc} \omega_{11} & \omega_{12} & \ldots & \omega_{1L}\\ \omega_{21} & \omega_{22} & \ldots & \omega_{2L}\\ \vdots & \vdots & \ddots & \vdots \\ \omega_{M1} & \omega_{M2} & \ldots & \omega_{ML} \end{array}\right)$$

Both azimuth and elevation angles are embedded in $w_{ik}$. Accordingly, the array steering vector corresponding to the *k*-th impinging signal can be expressed, following the 3GPP spatial channel modeling framework [41], as shown in Equation (11):

(11) $$a\left(\theta_{k},\phi_{k}\right)={\left[\begin{array}{cccc} e^{-jw_{1k}} & e^{-jw_{2k}} & \ldots & e^{-jw_{Mk}} \end{array}\right]}^{T}$$

## 3. Construction of the Projection Matrix

In order to obtain interpretable data summarization, the CSS problem has been used in numerous real-world applications [42]. The estimation accuracy is considerably influenced by the sampling process employed to extract specific columns from the original matrix. Consequently, an effective sampling strategy is essential for constructing a reduced-dimension matrix to enhance DoA estimation performance. To examine this issue and reassess the most pertinent concepts, imagine an array antenna consisting of M elements utilized to gather incident signals for N instances. The comprehensive data matrix obtained can be depicted as follows:

(12) $$X\left(t\right)=\left(\begin{array}{ccccc} x_{1}\left(t_{1}\right) & x_{1}\left(t_{2}\right) & \ldots & \ldots & x_{1}\left(t_{N}\right)\\ x_{2}\left(t_{1}\right) & x_{2}\left(t_{2}\right) & \vdots & \vdots & x_{2}\left(t_{N}\right)\\ \vdots & \vdots & \ddots & \vdots & \vdots \\ \vdots & \vdots & \ldots & \ddots & \vdots \\ x_{M}\left(t_{1}\right) & x_{M}\left(t_{2}\right) & \ldots & \ldots & x_{M}\left(t_{N}\right) \end{array}\right)$$

The predicted value of the outer product defines the real CM of the received signal, $R_{xx}$:

(13) $$R_{xx}=E\;\left[X\left(t\right)X^{H}\left(t\right)\right]=E\;\left[AS\left(t\right)S^{H}\left(t\right)A^{H}\right]+E\;\left[N\left(t\right)N^{H}\left(t\right)\right]=AR_{ss}A^{H}+\sigma_{n}^{2}I_{M}$$

Therefore, the source covariance matrix of size L×L is $R_{ss}=E\left[S\left(t\right)S^{H}\left(t\right)\right]$. $I_{M}$ is the identity matrix of size M×M, whereas $\sigma_{n}^{2}$ represents the variance of the noise. The variance of the noise is indicated by the term $\sigma_{n}^{2}$, and the identity matrix of size M×M is represented by $I_{M}$. The expectation operator is denoted by E[·] and the Hermitian (conjugate) transpose is represented by the notation ${\left(\cdot \right)}^{H}$. The signal and noise components are assumed to be independent in this formulation.

Since noise and signals are uncorrelated [42], the right-hand side equality in Equation (14) holds. As a sample-average, it can be calculated:

(14) $${\hat{R}}_{xx}=\frac{1}{N}\;XX^{H}=\left(\begin{array}{cccc} r_{11} & r_{12} & \ldots & r_{1M}\\ r_{21} & r_{22} & \ldots & r_{2M}\\ \vdots & \vdots & \ddots & \vdots \\ r_{M1} & r_{M2} & \ldots & r_{MM} \end{array}\right)$$

For each element $r_{i}$ in the calculated covariance matrix ${\hat{R}}_{xx}$, the spatial correlation between the signals received at the *i*-th and *j*-th antenna elements is recorded. Array signal processing makes extensive use of this statistical representation to estimate the spatial characteristics of the incoming wavefronts. To tackle the dimensionality issue, numerous structured and randomized sampling techniques have been suggested to create a lower-dimensional projection matrix Q, which selects *L* columns from ${\hat{R}}_{xx}$ or directly from X(t). In CS, US, and NUS, this sampling is generally implemented using fixed or randomly distributed indices.

### 3.1. Sampling Methodologies for Projection Matrix Construction

The three most common sampling methodologies used to construct a PM from a CM are Classical Sampling, Uniform Sampling, and Non-Uniform Sampling. It is important to note that each methodology has its own advantages and challenges, which impact the overall performance of the DoA estimation process.

#### 3.1.1. Classical Sampling Methodology (CSM)

The simplest and most natural method for creating the PM from the CM is the classical sampling method. This method, which was first presented in earlier research [31,32], chooses the first *L* columns of the sample ${\hat{R}}_{xx}$ and arranges them into a matrix $Q_{Class}$ as follows:

(15) $$Q_{class}=\left(\begin{array}{cccc} r_{11} & r_{12} & \ldots & r_{1L}\\ r_{21} & r_{22} & \ldots & r_{2L}\\ \vdots & \vdots & \ddots & \vdots \\ r_{M1} & r_{M2} & \ldots & r_{ML} \end{array}\right)$$

We can rewrite $Q_{Class}=\left[\begin{array}{cccc} c_{1} & c_{2} & \ldots & c_{L} \end{array}\right]$ based on the conventional technique. The sampled matrix $Q_{\mathrm{Class}}$ can be rewritten using the conventional sampling approach as follows: $Q_{\mathrm{Class}}=\left[c_{1}\;c_{2}\;\ldots \;c_{L}\right]$ where each $c_{i}$, for 1≤i≤L, corresponds to the sample ${\hat{R}}_{xx}$’s $i^{\mathrm{th}}$ column.

In this case, the matrix $Q_{\mathrm{Class}}\in C^{M\times L}$ is built so that its columns try to cover the same subspace as the actual signal components. The dominant subspace properties of the sampled matrix are preserved by this design assumption. The following expression illustrates how the PM based on the classical technique is created by projecting onto the orthogonal complement of the subspace spanned by $Q_{\mathrm{Class}}$:

(16) $$U_{Class}=I_{M}-Q_{Class}{\left(Q_{Class}^{H}Q_{Class}\right)}^{-1}Q_{Class}^{H}$$

In the spatial spectrum, the clear peak locations represent the directions from which the signals arrive, which are obtained using Equation (17).

(17) $${\left({\hat{\theta }}_{L},{\hat{\phi }}_{L}\right)}_{Class}=\arg\max_{\left(\theta_{L},\phi_{L}\right)}\left\{\frac{1}{{∥a\left(\theta ,\phi \right)U_{Class}∥}^{2}}\right\}$$

**Figure 2:**
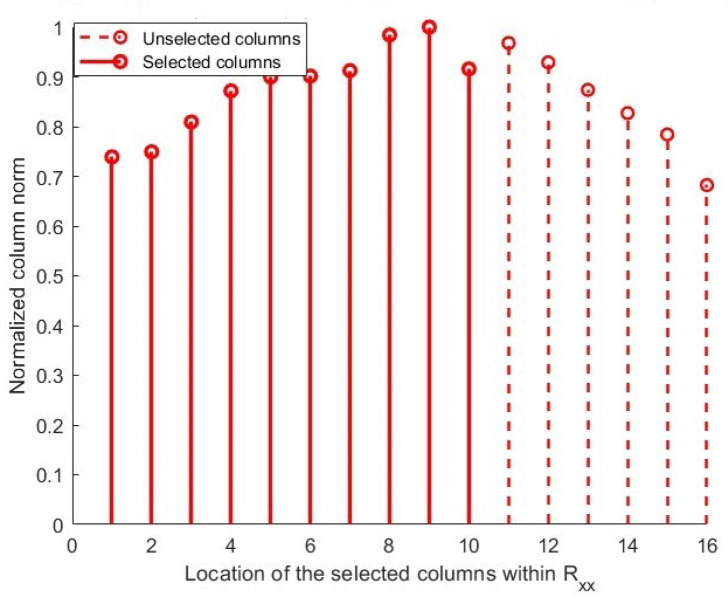
Location of selected columns within normalized columns using classical sampling.

As shown in Figure 2, sampling CM is implemented using a classical method to construct a sampled matrix. For all the methodologies considered, we plot the selected columns’ positions (x-axis) against the normalized column norm (i.e., y-axis), which is a direct representation of the correlation levels between incident signals within each column. In this approach, there is no need for complex computations or algorithms, which is its main advantage. However, this method has significant drawbacks. The classical selection technique derives the first L columns from the CM without assessing their structural importance or intercorrelations. This fixed selection diminishes the DOF and leads to inefficient utilization of the array aperture. Furthermore, the absence of inter-column correlation consideration frequently results in selected columns demonstrating significant linear dependence. This redundancy can result in the sampled matrix inadequately representing the true signal subspace, hence significantly impairing the accuracy of DoA calculation [31].

#### 3.1.2. Uniform Sampling Methodology (USM)

USM was developed to overcome some limitations of classical sampling methods, particularly in improving the degree of freedom. USM spreads selected columns more evenly across the CM instead of simply selecting the first L columns [29]. This formula determines their positions in accordance with a predetermined formula:

(18) $$Q_{USM}=\left\{c_{i}|c_{i}=1+\mathrm{round}{\left(\frac{\left(i-1\right)\left(M-1\right)}{L}\right)}_{i=1,2,\ldots ,L+1}\right\}$$

**Figure 3:**
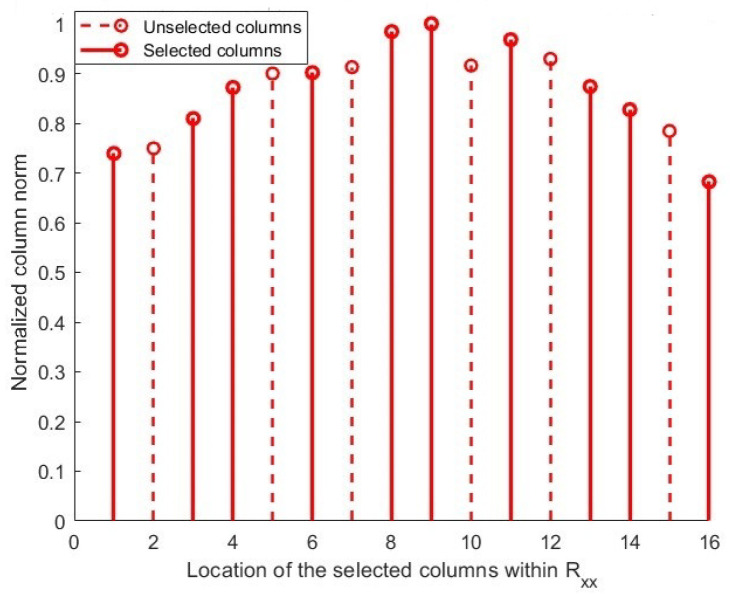
Location of selected columns within normalized columns using USM.

In Figure 3, the sampled matrix generated using the USM is depicted. While USM improves the DOF compared to conventional methods by more evenly distributing the selected columns across the array aperture, it still has notable limitations. Specifically, the locations of the selected columns remain non-adaptive and are predefined by the system, preventing the method from dynamically adjusting to the characteristics of the CM. Additionally, similar to the classical method, USM continues to select columns with high correlation coefficients (CCs), which can impair the effectiveness of the DoA estimation. Despite the increased DOF, these issues, particularly the persistent high correlation between columns, limit the potential improvements in estimation performance.

#### 3.1.3. Non-Uniform Sampling Methodology (NUSM)

NUSM is an advanced approach developed to enhance the performance of DoA estimation by focusing on the total energy of the signal eigenvalues. Unlike classical and uniform sampling methods that rely on predefined or systematic column selection from the CM, NUSM randomly selects columns using principles from random matrix theory and low-rank matrix approximation techniques [30,43]. This random selection process maintains the essential characteristics of the original matrix while effectively reducing its dimensionality, which helps preserve the signal’s integrity.

One of the key advantages of NUSM is its ability to improve noise immunity. By randomly distributing the selected columns, the total energy of the signal eigenvalues is increased, leading to a more robust DoA estimation process. This enhancement in signal energy directly contributes to improved performance in noisy environments, making the estimation algorithm more resilient against interference. While NUSM’s column locations are dynamic, the issue of picking high-CC columns remains unresolved. This is an important issue for both classical and USM, as well as NUSM. Once the sampled matrix QNUSM is created, the algorithm follows the conventional technique by replacing QClass with QNUSM and estimating DoAs using Equations (16) and (17).

**Figure 4:**
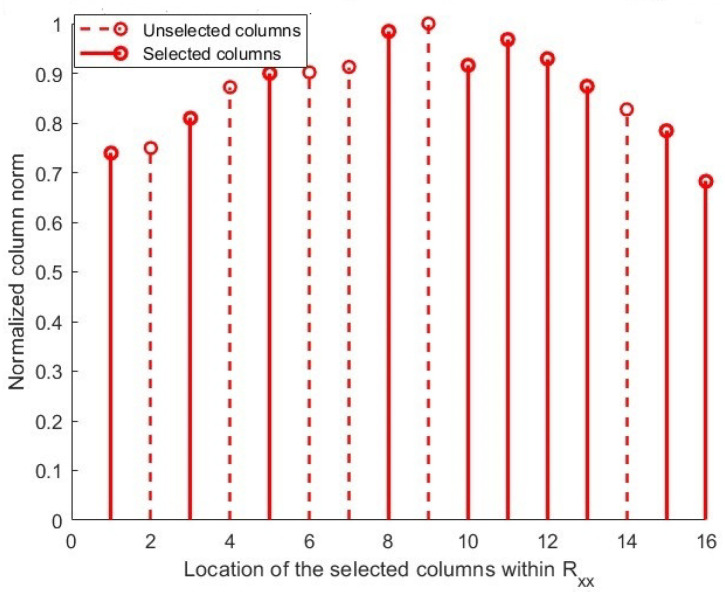
Location of selected columns within normalized columns using NUSM.

Figure 4 demonstrates that the QNUSM matrix has L randomly extracted columns from the CM. The NUS methodology selects columns without using criteria, such as high or low correlation between signals (e.g, column in Figure 4).

However, despite these improvements, NUSM still struggles with correlation issues. The random nature of column selection does not guarantee that columns with low CCs will be chosen. As a result, some selected columns may still have high correlations as shown in [43], which can have a negative impact on the accuracy and reliability of DoA estimation. This persistent issue indicates that while NUSM advances beyond classical methods, further refinement is needed to address the correlation problem effectively.

## 4. Reduced Angle Subspace Algorithm (RASA)

According to the comprehensive descriptions and conceptual representations in Figure 2, Figure 3 and Figure 4 of Section 3, a primary problem in DoA estimation is the heightened computing complexity and the correlation among the columns derived from the sample covariance matrix ${\hat{R}}_{xx}$.

These problems frequently result in a rank-deficient matrix Q, which restricts the estimate process’s accuracy and stability. By choosing the least redundant and most decorrelated columns from the noise subspace, the RASA is proposed and presented in this paper as a solution to this issue. The approach is appropriate for real-time or resource-constrained contexts because of this selection procedure, which also minimizes computational cost and reduces the dimensions of the subspace. Furthermore, RASA improves the estimation’s robustness in the presence of partially coherent signals, which frequently impair the performance of conventional subspace approaches, by concentrating on lowering inter-column correlation. In general, conventional methods such as CSM, USM, NUSM, Proposed LCCS, Proposed SS, and Real Proposed do not comprehensively address inter-column correlation and numerical conditioning, often leading to degraded performance under coherent or low-SNR conditions [29,30,31,32].

### 4.1. Correlation-Aware Column Selection in RASA

The RASA method uses a selective column sampling approach to reduce rank deficit and redundancy in sampled subspace matrices. This approach is founded on correlation-aware selection principles. The fundamental objective is to ensure that each selected column from the noise subspace offers unique information about the directions of incoming signals with minimum correlation. The technique initiates by calculating the sample covariance matrix ${\hat{R}}_{xx}$ from the received signal matrix $X\in C^{M\times N}$, where *M* denotes the number of sensors and *N* represents the number of snapshots:

(19) $${\hat{R}}_{xx}=\frac{1}{N}XX^{H}$$

The subsequent stage is performing eigen-decomposition on the estimated covariance matrix ${\hat{R}}_{xx}$ to derive its eigenvalues and eigenvectors, which are subsequently utilized to construct the signal and noise subspaces:

(20) $$R_{xx}=V\Lambda V^{H}$$

where:

- $\Lambda \in R^{M\times M}$ is the diagonal matrix of eigenvalues, arranged in descending order.
- $V\in C^{M\times M}$ is the eigenvector matrix.

From this decomposition, we obtain the noise subspace matrix $Q_{n}\in C^{Mx(M-L)}$, comprising the eigenvectors corresponding to the smallest M−L eigenvalues:

(21) $$Q_{n}=\left[v_{L+1},v_{L+2},\ldots ,v_{M}\right]$$

Here, $v_{L+1},v_{L+2},\ldots ,v_{M}$ represent the eigenvectors of the covariance matrix $R_{xx}$ associated with the smallest (M−L) eigenvalues, together constituting the noise subspace matrix $Q_{n}$.

To achieve effective decorrelation and preserve orthogonality across subspace components, the correlation matrix $C_{n}$ is calculated using the Pearson correlation coefficient applied to the real-valued portion of the noisy subspace matrix $Q_{n}$. This is articulated as:

(22) $$C_{n}=\mathrm{corrcoef}\left(ℜ\left(Q_{n}\right)\right)$$

In Equation (22), only the real component of $Q_{n}$ is utilized, as the Pearson correlation coefficient is applicable solely to real-valued variables, and its direct application to complex entries may produce ambiguous outcomes. Limiting the procedure to $ℜ(Q_{n})$ yields restricted and interpretable correlation values, while empirical studies validated that this selection maintains the fundamental inter-column structure without compromising subspace information.

To measure the overall correlation strength of each column $c_{i}$ in $C_{n}$, we assess its $\ell_{2}$-norm, which indicates the total correlation of column *i* with the remaining subspace. This is mathematically defined as:

(23) $$\gamma_{i}={∥C_{n}\left(:,i\right)∥}_{2}=\sqrt{\sum_{j=1}^{M-L}{\left(C_{ji}\right)}^{2}},\;i=1,\ldots ,M-L$$

The RASA algorithm uses this metric $\gamma_{i}$ to facilitate resilient subspace selection by identifying and ranking columns with low redundancy. It gives a scalar measure of the correlation density for each column. Less redundant and more decorrelated columns have a lower $\ell_{2}$-norm.

In order to improve the selection process even further, $\ell_{2}$-norm are standardized in the following ways:

(24) $${\tilde{\gamma }}_{i}=\frac{\gamma_{i}}{\max_{k}\gamma_{k}}$$

The normalization function in Equation (24) rescales $\gamma_{i}$ by dividing it by the maximum of all $\gamma_{k}$ values, where *k* represents the set of elements. Here, $\gamma_{i}$ is a scalar metric (e.g., norm or correlation) for the *i*-th column, and $\max_{k}\gamma_{k}$ is the maximum value of all such metrics. The normalized score ${\tilde{\gamma }}_{i}$ falls inside the range [0,1], enabling fair comparison and ranking across all columns.

The first and last columns are not taken into account while choosing a subspace in order to reduce boundary effects. Therefore, the following defines the candidate subset:

(25) $$J_{c}=\left\{2,3,\ldots ,M-L-1\right\}$$

The index set of candidate columns, $J_{c}$, ranges from the second to the (M−L−1)-th column, eliminating border columns to avoid edge effects. Then, extract the norms of the subset:

(26) $$\Gamma_{J_{c}}=\left(\gamma_{i}|i\in J_{c}\right)$$

The set $\Gamma_{J_{c}}$ contains scalar values $\gamma_{i}$ for select column indices $i\in J_{c}$ for evaluation. The reduced set of minimally correlated interior columns is formed by sorting $\Gamma_{J_{c}}$ in ascending order and selecting the smallest L−2 values for inclusion in the final subspace.

The RASA algorithm selects the following subspaces in order to construct a spatially informative and low-redundancy subspace:

- The initial and final $Q_{n}$ columns.
- The L−2 columns from the interior subset $J_{c}=\left\{2,3,\ldots ,M-L-1\right\}$ that have the fewest $\ell_{2}$-norm.

The following subset is extracted:

(27) $$J=argsort_{i\in J_{c}}\left(\gamma_{i}\right)\left[1:\left(L-2\right)\right]$$

In the end, the following set of indices was selected:

(28) $$J^{*}=\left\{1\right\}\cup J\cup \left\{M-L\right\}$$

In order to maintain aperture diversity, prevent degenerate subspaces, and guarantee that no crucial information of $Q_{n}$ is lost, the first and last columns in Equation (28) are deterministically included to make up for their previous exclusion during ranking. As a result of these indices, we are able to construct the reduced noise subspace matrix according to Equation (29).

(29) $$Q_{RASA}=Q_{n}\left(:,J^{*}\right)\in C^{MxL}$$

**Figure 5:**
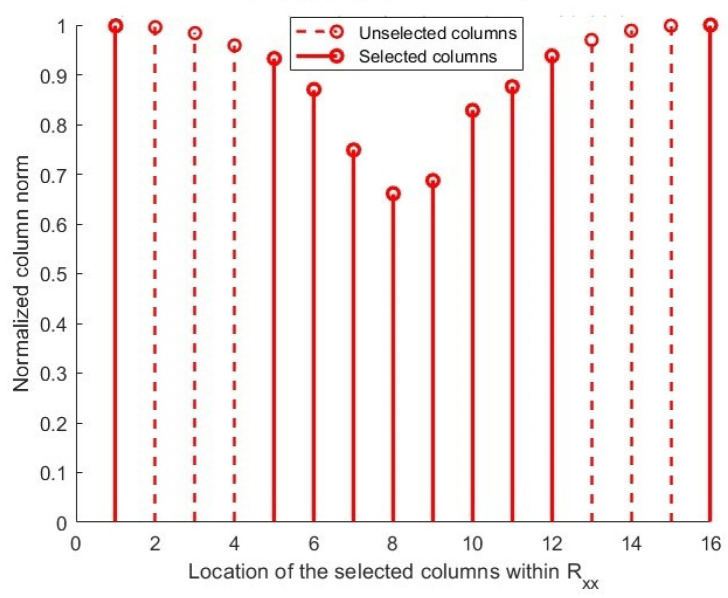
Location of selected columns within normalized columns using LCCS.

This condensed subspace enhances the DoA estimation performance under high noise and signal coherence while preserving the most informative, minimally correlated elements of the noise subspace. Figure 5 shows that the signals are least associated with unselected columns. The suggested method uses columns with the lowest CCs (represented by column norm) to generate the sampling matrix, as depicted in the image [44].

A further refinement is necessary to enforce structural decorrelation and guarantee representation uniqueness, even though the $\ell_{2}$-norm-based approach mentioned above guarantees the selection of minimally correlated columns from the noise subspace. The following part addresses this by introducing a structured anti-correlated subspace approximation approach that uses normalized correlation metrics to systematically analyze the pairwise inter-column dependencies.

### 4.2. Reduced and Structured Anticorrelated Subspace Approximation RASA Methodology

This section introduces a systematic modification to column selection within the noise subspace to retain array aperture and increase subspace decorrelation. By using their $\ell_{2}$-norm to identify minimally correlated columns, the suggested method makes sure that every component chosen transmits unique signal information with the least amount of redundancy. A more robust and illuminating subspace representation is made possible by measuring inter-column dependency inside the sampled noise matrix $Q_{n}$.

This strategy uses CM-based selection, where normalized correlation norms measure column similarity to all others. Lower correlation norms suggest more independent columns, improving DoA resolution in low SNR or coherent signal situations. The least dependent columns can be found practically by calculating the CM of Q and choosing the columns with the smallest normalized correlation norms. The correlation between one column and every other column is represented by the norm of each column. Weaker inter-sensor correlation is shown by lower norm values because the CC is a unitless measure that is formed from off-diagonal CM inputs. As a result, only when a column shows little correlation with the other columns in the matrix is it chosen.

This procedure ensures that the chosen columns retain subspace uniqueness by reducing the correlation between $X_{i}$ and $X_{\left(j\neq i\right)}$. Since each column in the sampled matrix represents a different signal direction, RASA satisfies the unique representation property [37].

The RASA algorithm chooses the remaining columns from the intermediate set according to their decorrelation efficiency, always including the initial and last columns to maintain aperture and edge variety. The expression for the resulting submatrix is expressed in Equation (30). In this case, *c* represents the column index set defined by:

(30) $$c=\left\{Z|Z=2,3,\ldots ,M-1\right\}⟶Q_{RASA}=\left[\vec{r_{1}}\;\vec{r_{c1}}\;\vec{r_{c2}}\;\ldots \;\vec{r_{c_{L_{2}}}}\;\vec{r_{M}}\right]$$

To eliminate boundary bias, Z={2,3,…,M−1} represents all intermediate column indices except the first and last. The matrix $Q_{\mathrm{RASA}}$ is created by picking a subset of columns from the noise subspace matrix, where ${\vec{r}}_{j}$ represents the *j*-th column vector of $Q_{n}$. The RASA criterion identified ${\vec{r}}_{c_{1}},{\vec{r}}_{c_{2}},\ldots ,{\vec{r}}_{c_{L_{2}}}$ as the least correlated columns. The whole set of indices, encompassing the M−L unselected columns denoted by $c_{u}$ and the L−2 selected columns denoted by $c_{s}$, is specifically represented by the letter *c*. These subsets of *C* have the properties ∣c∣=M−2 and $c=c_{s}\cup c_{u}$. Finding the L−2 intermediate columns once the first and last are fixed is the primary challenge.

When choosing columns, RASA expressly minimizes the correlation between signals obtained at various sensor elements, in contrast to uniform [29] or random [30] selection procedures. In particular, it selects $c_{s}$ to minimize $corr(X_{i},X_{j\neq i})$.

It is quantified by utilizing the $\ell_{2}$-norm of each column of CM to quantify the total signal correlation. In order to select the most decorrelated columns, the algorithm uses the following criteria:

(31) $$c=\left\{\begin{array}{c} c_{s},{∥r_{c_{s}}∥}_{2}=\min\left\{{∥r_{c}∥}_{2}\right\}\;\mathrm{for}\;1\leq s\leq L-2\\ c_{u},{∥r_{c_{u}}∥}_{2}=\max\left\{{∥r_{c}∥}_{2}\right\}\;\mathrm{for}\;1\leq u\leq M-L \end{array}\right.$$

In this case, ${\vec{r}}_{c}$ is a generic column vector from the noise subspace matrix $Q_{n}$. The index *s* represents a selected column with minimal $\ell_{2}$ norm, while *u* represents an unselected column with maximal norm. The vectors ${\vec{r}}_{c_{s}}$ and ${\vec{r}}_{c_{u}}$ indicate the energy level of the most and least dominant columns in their subsets, and the $\ell_{2}-norm$ of each CM column is indicated by ${\left\|\cdot \right\|}_{2}$. The lowest and highest norm values are returned by the min and max functions in turn. Equation (31) is another way to restate this rule. Here, Position returns the relevant indices and mink(·) (The function mink(A,k) returns a row vector **B** that contains the **k** smallest elements from the array **A**. In the context of our method, we define $A=\{\|\gamma c{\|}_{2}\in R^{1xM-2}\}$, where k=L−2. Similarly, the mask function can be used to retrieve the *k* largest elements from the same set $\left\{\|\gamma c{\|}_{2}\right\}$) and maxk(·) yield the norm set’s smallest L−2 and greatest M−L values, respectively.

(32) $$\begin{array}{c} c_{s}=\mathrm{Position}\left(\left[mink\left(\left\{{∥r_{c}∥}_{2}\right\},L-2\right)\right]\right)\\ c_{u}=\mathrm{Position}\left(\left[maxk\left(\left\{{∥r_{c}∥}_{2}\right\},M-L\right)\right]\right) \end{array}$$

The sample matrix $Q_{\mathrm{RASA}}$ is created using deterministic and decorrelated columns, selected using Equations (31) and (32). The first column with the lowest $\ell_{2}$ -norm is picked, followed by the L−2 least correlated columns from the remaining contenders. Finally, the final column in $Q_{\mathrm{RASA}}$ has the highest $\ell_{2}$-norm. The selected subspace has maximum angular resolution and the least redundancy with this hierarchical design.

The following is a summary of the two primary benefits of the suggested RASA sampling method. By purposefully including the first and last columns, $\vec{r_{1}}$ and $\vec{r_{M}}$, in the created matrix, it initially optimizes the array aperture. Because of this design, signals coming from extreme angular directions at the array edge can be efficiently captured by the RASA based on DoA estimation. Secondly, it reduces the correlation between adjacent columns in the matrix that was sampled. This feature is crucial for resolving multiple signal directions since it guarantees that the chosen subspace columns have non-redundant spatial information. This improves angular resolution beyond what is possible with current subspace sampling techniques by using decorrelated and geographically varied subspace components, which guarantees higher overall performance.

### 4.3. Theoretical Evaluation of Subspace Structure Through Adjacent Column Analysis Based on Correlation

Initially, the Pearson Product Moment Correlation Coefficient (PPMCC) was used as the starting point for analysis in order to quantify the degree of the strength correlation between adjacent columns. The PPMCC, represented by η, can be used to characterize the relationship between neighboring columns within *Z*, such as $Z_{k}$ and $Z_{k+1}$, and can be calculated using Equation (33).

(33) $$\eta_{z_{k},z_{k+1}}=\frac{\sum_{k=1}^{L-1}\left(z_{k}-{\bar{z}}_{k}\right)\left(z_{k}-{\bar{z}}_{k}\right)\left(z_{k+1}-{\bar{z}}_{k+1}\right)}{\sqrt{\sum_{k=1}^{L-1}{\left(z_{k}-{\bar{z}}_{k}\right)}^{2}\sum_{k=1}^{L-1}{\left(z_{k+1}-{\bar{z}}_{k+1}\right)}^{2}}}$$

The mean values of ${\bar{z}}_{k}$ and ${\bar{z}}_{k+1}$ are represented by $z_{k}$ and $z_{k+1}$, respectively, and are calculated as follows:

(34) $${\bar{z}}_{k}=E\left(z_{k}\right)=\sum_{k=1}^{L-1}z_{k}P\left(z_{k}\right),\;{\bar{z}}_{k+1}=E\left(z_{k+1}\right)=\sum_{k=1}^{L-1}z_{k+1}P\left(z_{k+1}\right)$$

In this case, P(·) represents the likelihood of choosing the *n*-th column. The symbols $z_{k}$ and $z_{k+1}$ represent the mean-adjusted values of ${\hat{z}}_{k}$ and ${\hat{z}}_{k+1}$, respectively. Therefore, another way to represent $\eta_{z_{k},z_{k+1}}$ is as follows:

(35) $$\rho_{z_{k},z_{k+1}}\in R^{L-1}=\frac{\sum_{k=1}^{L-1}\left({\hat{z}}_{k}\right)\left({\hat{z}}_{k+1}\right)}{\sqrt{\sum_{k=1}^{L-1}{\left({\hat{z}}_{k}\right)}^{2}\sum_{k=1}^{L-1}{\left({\hat{z}}_{k+1}\right)}^{2}}}$$

According to the Cauchy–Schwarz inequality, the following is accurate:

$$|{\hat{z}}_{1}{\hat{z}}_{2}+{\hat{z}}_{2}{\hat{z}}_{3}+\ldots +\left|{\hat{z}}_{L-1}{\hat{z}}_{L}\right|\leq \sqrt{{\left({\hat{z}}_{1}\right)}^{2}+{\left({\hat{z}}_{2}\right)}^{2}+\ldots +{\left({\hat{z}}_{L-1}\right)}^{2}}\times \sqrt{{\left({\hat{z}}_{2}\right)}^{2}+{\left({\hat{z}}_{3}\right)}^{2}+\ldots +{\left({\hat{z}}_{L}\right)}^{2}}$$

Thus, it follows logically that the PPMCC range is $0\leq |\rho_{z_{k},z_{k+1}}|\leq 1$. The value $|\rho_{z_{k},z_{k+1}}|$ degree of linear relationship between $z_{k}$ and $z_{k+1}$ is represented by the normalized measure [45]. Perfect correlation is represented by a value of 1, and no correlation is represented by a value of 0. It is implied that the two vectors carry independent information when the coefficient is zero. On the other hand, total redundancy is indicated by a value of 1. In this situation, $|\rho_{z_{k},z_{k+1}}|$ is reduced to as close to zero as possible.

To quantify inter-column correlation, the Pearson Product Moment Correlation Coefficient (PPMCC) was computed as in Equation (33) and Equation (35). Table 1 reports the mean absolute adjacent-column correlations for each sampling strategy. Conventional methods (CSM, USM, NUSM) exhibited consistently high correlation values (≈0.10–0.15), indicating strong redundancy. LCCS reduced correlation substantially, while the RASA method achieved the lowest values (≈0.05–0.09), demonstrating superior decorrelation. To validate the proposed strategies, a uniform linear array (ULA) with M=30 sensors was considered under coherent source conditions. The covariance matrix of size 30×30 was estimated using N=100 snapshots at an SNR of 0 dB. To ensure statistical robustness, K=1000 Monte Carlo trials were conducted, with each trial selecting L=10 columns.

To confirm the statistical robustness of RASA’s decorrelation improvement, paired trial values were subjected to the Wilcoxon signed-rank test [46]. The difference between a baseline value $x_{i}$ and the accompanying RASA value $y_{i}$ for *N* paired trials can be expressed as $d_{i}=x_{i}-y_{i}$. Equation (36) provides the definition of the signed-rank statistic.

(36) $$R^{+}=\sum_{d_{i}>0}\mathrm{rank}(|d_{i}|),\;R^{-}=\sum_{d_{i}<0}\mathrm{rank}(|d_{i}\left|\right),\;T=\min\left(R^{+},\;R^{-}\right)$$

In this case, *T* is the Wilcoxon statistic, and $R^{+}$ and $R^{-}$ are the sums of ranks of the positive and negative differences. For the signed-rank test, Equation (36) ranks positive and negative paired differences.

The distribution of *T* can be roughly represented by a normal distribution for large sample sizes (N=1000 trials). This enables the significance level to be directly calculated using a *z*-score. Equation (37) provides the typical approximation used in this work.

(37) $$z=\frac{T-\frac{1}{4}N\left(N+1\right)}{\sqrt{\frac{1}{24}N\left(N+1\right)\left(2N+1\right)}},$$

Since RASA is evaluated against many baselines, the *p*-values were adjusted using the Holm–Bonferroni procedure [47]:

(38) $$p_{i}^{\mathrm{Holm}}=\min\left(1,\max_{j\leq i}\left\{\left(m-j+1\right)\;p_{\left(j\right)}\right\}\right),$$

where the sorted raw *p*-values from *m* comparisons are indicated by $p_{\left(j\right)}$. The family-wise error rate is rigorously controlled by Equation (38), avoiding overblown significance brought on by repeated testing. The Wilcoxon signed-rank [46] test results are summarized in Table 2 to ensure these reductions are not due to chance. In comparison to CSM, USM, and NUSM, RASA lowers redundancy, as indicated by negative DeltaMedian values; nonetheless, positive DeltaMedian values against LCCS confirm further improvement beyond its partial elimination. The huge effect sizes (r>0.65) and the fact that all *p*-values are well below 0.05 show that the improvement is both practically and statistically meaningful. Together, Table 1 and Table 2 confirm that RASA generates decorrelated and structurally robust subspaces, which are essential for accurate DoA estimation.

In comparison to baseline approaches (RASA was used as the reference technique in Wilcoxon signed-rank analysis, and all differences are measured relative to it. The table does not have a distinct row for RASA because each baseline is directly compared to it), RASA consistently produces decorrelation improvements that are statistically significant and practically meaningful, as shown by this dual descriptive–inferential evaluation (Table 1 and Table 2).

### 4.4. Application of RASA for High-Resolution DoA Estimation

To improve spatial spectrum estimation and DoA performance, the reduced noise-subspace matrix $Q_{\mathrm{RASA}}\in C^{M\times r}$ with r≤M−L, formed by selecting the least-correlated columns from the estimated noise subspace $Q_{n}$, is inserted into the conventional MUSIC framework.

The steering vector for an angle θ is given by:

(39) $$a\left(\theta \right)=\exp\left(j2\pi d\cdot u\cdot \sin(\theta )\right),\;u={\left[0,1,\ldots ,M-1\right]}^{T}$$

In this case, *d* represents the inter-element spacing (typically λ/2), u represents the sensor index vector for a ULA, and θ∈Θ represents the angle search space, where Θ includes values like $\left[-90^{\circ },90^{\circ }\right]$. After that, the pseudo-spectrum of RASA-enhanced MUSIC is calculated as Equation (37):

(40) $$P_{\mathrm{RASA}}\left(\theta \right)=\frac{1}{a^{H}\left(\theta \right)Q_{\mathrm{RASA}}Q_{\mathrm{RASA}}^{H}a\left(\theta \right)}$$

Next, the estimated directions of arrival are determined by:

(41) $${\hat{\theta }}_{\mathrm{RASA}}=\arg\max_{\theta \in \Theta }\left\{10\log_{10}\left(P_{\mathrm{RASA}}\left(\theta \right)\right)\right\}$$

This approach replaces the full MUSIC projector $Q_{n}Q_{n}^{H}$ with the reduced projector $Q_{\mathrm{RASA}}Q_{\mathrm{RASA}}^{H}$, constructed from the least-correlated columns, offering the following advantages: Sharper spectral peaks, enhanced angular resolution, reduced computational complexity due to subspace compression, and improved robustness in the presence of noise and coherent signals. This RASA implementation successfully converts the conventional MUSIC framework into a high-resolution, low-complexity estimator appropriate for real-world situations involving array defects, redundancy, or interference. The algorithm steps are detailed in Algorithm 1.
**Algorithm** **1:** Steps of the RASA Algorithm**Input:** Received data $X\in C^{M\times N}$ at the output of an array composed of *M* antennas, with *N* snapshots, and *L* incident signals**Output:** Accurate DoAs Estimation**1** **Step** **1:** Calculate the sample covariance matrix ${\hat{R}}_{xx}=\frac{1}{N}\left(XX^{H}\right)$**2** **Step** **2:** Perform eigen decomposition on ${\hat{R}}_{xx}$ to obtain eigenvectors *Q* and eigenvalues Λ, and sort them in descending order of eigenvalues**3** **Step** **3:** Identify the noise subspace $Q_{n}$ by selecting the eigenvectors corresponding to the smallest M−L eigenvalues**4** **Step** **4:** Compute the correlation matrix $C=\mathrm{corrcoef}(Q_{n})$**5** **Step** **5:** Calculate the $\ell_{2}$-norms for each column of *C*, excluding the first and last columns**6** **Step** **6:** Sort the $\ell_{2}$-norms in ascending order and select the L−2 columns with the smallest norms**7** **Step** **7:** Construct the reduced noise subspace matrix $Q_{LCCS}$ by sampling the first column, the last column, and the selected columns from Step 6 **8** **Step** **8:** Compute the MUSIC spectrum:$$P_{\mathrm{RASA}}\left(\theta \right)=\frac{1}{a^{H}\left(\theta \right)Q_{\mathrm{RASA}}Q_{\mathrm{RASA}}^{H}a\left(\theta \right)}.$$**Step** **9:** Identify the peaks of the MUSIC spectrum to estimate the DoAs

## 5. Comparative Analysis of Direction of Arrival Estimation Algorithms

This section provides a comparative analysis of DoA estimation algorithms based on a subspace, emphasizing their computational efficiency, robustness to noise, and spatial resolution. The algorithms considered include the Proposed LCCS, Proposed SS, USM, NUSM, CSM [45,48], and the RASA algorithm proposed in this paper.

Conventional CSM techniques employ the complete or fixed portions of the noise subspace without taking signal correlation into account, often resulting in redundancy, poor decorrelation, and reduced resolution. In contrast, USM and NUSM use random and uniform sampling techniques, respectively, to try to enhance this. While these methods increase aperture diversity, they frequently have weak durability or instability in coherent or low SNR settings [29].

In addition, the reduced-complexity MUSIC technique avoids eigen-decomposition through real-valued translation, making it applicable to all array types and reducing processing power. The increased spatial smoothing method refines the covariance matrix using intra- and inter-subarray correlations to improve DoA estimation accuracy under low SNR and snapshot situations. These methods are complementary and address distinct DoA estimation trade-space aspects.

In order to strike a balance between computational efficiency and estimation performance, numerous sophisticated methodologies have been proposed. The RASA algorithm is one such approach, which employs correlation and a first-last and least correlated column sampling technique to select a reduced yet informative noise subspace. In order to identify minimally correlated noise subspace vectors, the matrix $Q_{RASA}$ implements correlation-aware selection, which simplifies numerical conditioning but additionally reduces complexity.

### Comparison of Execution Times and Computational Complexity

**Figure 6:**
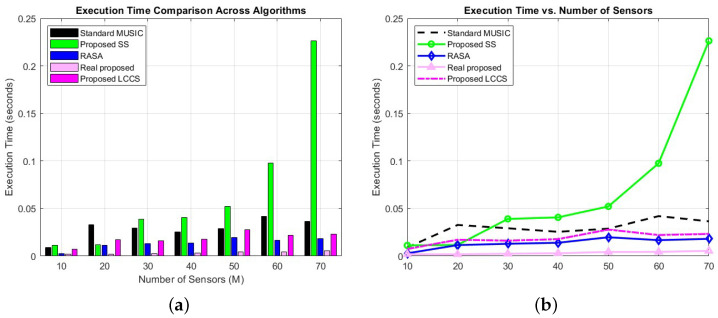
(**a**) Comparison of the execution times of the Real Proposed, Proposed-LCCS, Standard MUSIC, RASA, and Proposed SS algorithms for varying sensor counts. (**b**) Each algorithm’s computational efficiency and scalability as the number of sensors rises.

A comprehensive evaluation of the Standard MUSIC, RASA, Real Proposed, Proposed-LCCS, and Proposed SS algorithms was conducted to assess the computational efficiency of the proposed methods. Although USM and NUSM exhibit low to moderate complexity, they are excluded from the execution time comparison because RASA demonstrates substantially superior resolution performance. Therefore, the focus of this evaluation is to compare RASA with algorithms of comparable computational efficiency, such as the Real Proposed and Proposed SS methods, to provide an accurate assessment of execution times among these approaches. A series of experiments was conducted across five scenarios, varying the number of sensors (*M*). The setup was as follows: N=100 snapshots, SNR=0dB, λ=1, and scanning angles $\theta \in [-90^{\circ },90^{\circ }]$ with $0.1^{\circ }$ resolution. A total of L=10 sources were placed at uniformly distributed directions between $-90^{\circ }$ and $90^{\circ }$, all with $\phi =0^{\circ }$. The number of sensors was varied as M∈{10,20,30,40,50,60,70}. For the spectrum evaluation, each point represents MATLAB R2024b tic/toc timing. The distance between elements in the array was fixed at 0.5 wavelengths. Figure 6 displays the execution times of each method, which were measured by an AMD Ryzen 74 800H (2.9GHz) processor.

A comparison of the execution times of five algorithms with different numbers of array sensors is shown in Figure 6a,b. The measured execution times demonstrate the effectiveness of both the Real Proposed and RASA techniques. The runtimes of Standard MUSIC increase steadily as *M* increases because it scans the whole (M−K)-dimensional noise subspace and performs a full M×M eigenvalue decomposition (EVD). While RASA uses the same EVD stage, it lowers the scan cost and saves a significant amount of time by reducing the scan subspace to L+2 columns chosen using the $Q_{\mathrm{RASA}}$ criterion. Real Proposed attains the minimal execution durations for the majority of *M* values owing to its real-valued formulation and half-field-of-view (FOV) scan, which diminishes both the guiding vector dimension and the scanning range.

Nonetheless, for bigger arrays (M≥60), the advantages of RASA in diminished scan dimensions become increasingly apparent, and its runtime either approaches or somewhat exceeds that of Real Proposed. The proposed SS demonstrates significantly increased runtimes, especially for large *M*, owing to the repetitive calculations of subarray covariance and numerous P×P EVD operations. The Proposed LCCS also benefits from fixed-column projection, yielding competitive timings, albeit still above those of the two top algorithms.

In general, RASA and Real Proposed yield substantial runtime reductions in comparison to conventional methods, with the relative benefit contingent upon array size.

Among the evaluated methods, RASA and the Proposed Real-Value algorithm demonstrate practical advantages in terms of execution time. RASA achieves this through a streamlined subspace selection strategy that avoids expensive operations, while the Proposed Real-Value method benefits from its reduced matrix processing structure. In contrast, Proposed SS involves additional transformations and smoothing operations, resulting in increased computational time, particularly for RASA and the Real Proposed, for real-time or large-scale array processing systems, where both speed and accuracy are critical. These algorithms are highly suited for contemporary signal processing applications due to their ability to achieve a practical equilibrium between direction-finding precision and execution speed.

As can be seen in Table 3, the Floating Point Operation (FLOP) count provides a direct evaluation of computing cost by quantifying the theoretical FLOP needed by each algorithm. The parameters defined as follows, the number of sensors in the array is *M*, the number of sources or signals is *L*, the number of selected columns used for subspace construction is *J*, the size of the augmented matrix in the Proposed SS method is *P*, and the smoothing window or overlapping segments is *S*. Usually, parameters follow the relationships M>L, J≤M−L, and P>M. Structural selection algorithms that retain boundary columns and pick decorrelated or low-norm intermediate vectors shrink RASA and Proposed LCCS subspaces to L+2. Besides subspace size, difficulty classification depends on operations like sorting, projection, and inversion, which influence the algorithm’s processing requirement. Furthermore, the Real-Proposed technique and RASA’s reduced FLOP orders demonstrate their appropriateness for real-time, resource-constrained applications. On the other hand, approaches that include more processing steps, like Standard MUSIC and Proposed-SS, result in greater FLOP counts because of subspace manipulation and correlation-aware selection. While RASA and Proposed LCCS can operate on a subspace of dimension L+2, their computational architectures differ dramatically. Without matrix inversion and with decreased data, the RASA algorithm uses correlation norms and simple sorting in the noise subspace to choose columns. Proposed LCCS chooses columns straight from the complete covariance matrix based on their norms, then builds a projection matrix using a matrix inverse and several matrix multiplications. Adding operations, such as $O(L^{3})$ matrix inversion and $O(M^{2})$ matrix-vector products, increases computing cost. Overall, the FLOP analysis supports the usefulness of low-complexity methods in scalable DoA estimation and enhances empirical timing results.

## 6. Simulation Results and Performance Analysis of RASA

To demonstrate applicability across arbitrary array geometries, ageneric steering vector formulation is adopted, ensuring compatibility with both Uniform Linear Arrays (ULAs) and Uniform Rectangular Arrays (URAs). For illustration, initial results are presented for the URA, which supports two-dimensional scanning in azimuth and elevation due to its uniform element spacing along horizontal and vertical axes. Subsequent analysis focuses on the ULA configuration, which forms the basis of the main simulations. Performance is assessed under varied SNR levels, array sizes, correlated sources, and snapshot counts. The evaluation relies on the Average Root Mean Square Error (ARMSE) in (42) and the Probability of Successful Detection (PSD) in (43), providing consistent benchmarking across all considered array geometries. Here, *K* is the number of Monte Carlo trials, $NSD_{i}$ is the number of successful detections at the *i*-th simulation, and $\theta_{k}$ and ${\hat{\theta }}_{k}$ are the true and estimated DoAs at the *k*-th Monte Carlo iteration, respectively.

(42) $$\mathrm{ARMSE}=\frac{1}{K}\sum_{i=1}^{K}\sqrt{\frac{1}{L}\sum_{k=1}^{L}\left[{\left(\theta_{k}-{\hat{\theta }}_{k}\right)}^{2}\right]}$$

(43) $$PSD\left(DoAs\right)=\frac{\sum_{i=1}^{K}NSD_{i}}{KL}$$

### 6.1. Two-Dimensional (2D) DoA Estimation

To assess the effectiveness of the proposed DoA method with a 2D array configuration, we apply the URA with *M* elements positioned in the x–y plane. The goal is to estimate elevation angle θ and azimuth angle ϕ within the ranges $\left[0^{\circ },90^{\circ }\right]$ and $\left[0^{\circ },360^{\circ }\right]$, respectively. For this, we use a rectangular array with twelve equally spaced elements (M=12) to capture randomly generated BPSK signals transmitted by far-field sources. The simulation parameters are set as follows: SNR = 10 dB, element spacing d=0.5λ, and number of snapshots N=100. In this setup, four closely spaced plane waves (L=4) impinge on the array with elevation and azimuth angles given by: $\theta =[20^{\circ },22^{\circ },36^{\circ },39^{\circ }]$ and $\phi =[80^{\circ },25^{\circ },40^{\circ },100^{\circ }]$.

**Figure 7:**
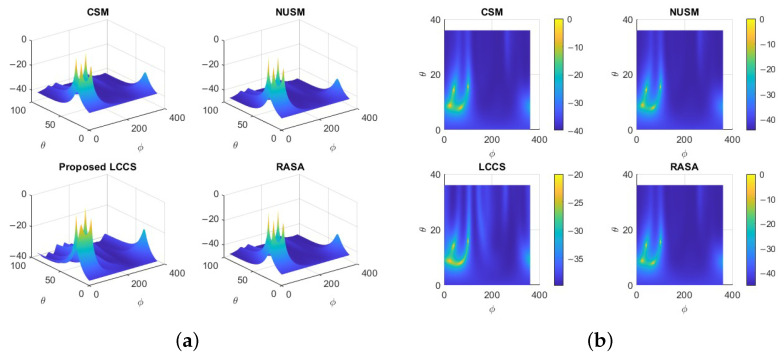
Estimation resolution of the RASA method for detecting (**a**) both the elevation and azimuth angles of four incident-signals clustered closely together, and (**b**) a top view of these signals.

In Figure 7a, all algorithms have identical peak locations, but a comparison of 3D RASA has sharper, more concentrated peaks with lower sidelobe levels than CSM and NUSM. Even with modest visual variations, these traits improve angular resolution and target separation. In multi-source circumstances, RASA’s noise subspace refinement and decorrelation improve peak sharpness and reduce ambiguity. As seen from above, the detected DoAs can be depicted as shown in Figure 7b. Despite the few visual variations, the RASA spectrum shows better resolution than other techniques, with less dispersion and sidelobe interference and a greater concentration around the correct angles. A comparison of 3D and 2D MUSIC-like spectra shows that the RASA yields the most compact mainlobes with reduced sidelobe leakage, making the peaks more clearly distinguishable than CSM, NUSM, and Proposed LCCS.

### 6.2. Performance on Non-Uniform Arrays

The algorithms’ performance under various sensor geometries was investigated by taking into account both two-dimensional non-uniform rectangular arrays and one-dimensional non-uniform linear arrays.

**Figure 8:**
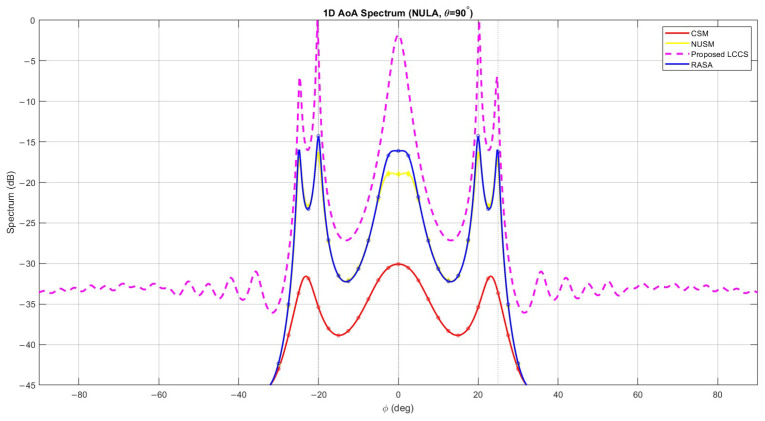
Azimuth pseudo-spectra for the 1D NULA case, where the RASA produces sharp peaks while CSM, NUSM, and Proposed LCCS show degraded responses.

The simulation used N=100 snapshots with an input SNR of 10 dB to replicate a NULA with M=32 unevenly spaced elements. Azimuths $-20^{\circ }$, $0^{\circ }$, and $+25^{\circ }$ were assigned to three uncorrelated sources at broadside elevation ($\theta =90^{\circ }$). Figure 8’s azimuth spectra demonstrate that CSM generates broad, poorly resolved peaks, whereas NUSM provides shallow responses that are unable to distinguish between the sources. The higher noise level and spurious lobes introduced by the proposed LCCS suggest that linear apertures have poor decorrelation capabilities. At the proper azimuths, the RASA generates sharp, well-localized peaks; however, because linear arrays are ambiguous on the left and right, the spectra display mirror symmetry, giving rise to five apparent peaks ($\pm 20^{\circ }$, $\pm 25^{\circ }$, and $0^{\circ }$) rather than three.

**Figure 9:**
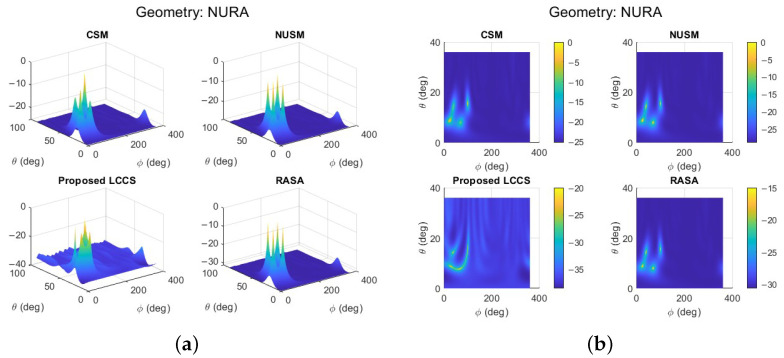
Pseudo-spectra for the 2D NURA case: (**a**) 3D representation and (**b**) top-down view, where the RASA provides accurate localization while the other methods show degraded or shifted responses.

A NURA comprising M=35 elements was simulated on a perturbed 7×5 grid with N=100 snapshots at an input SNR of 10 dB. Four sources were located at coordinates $\left(\theta ,\phi \right)=\left(20^{\circ },70^{\circ }\right)$, $\left(22^{\circ },25^{\circ }\right)$, $\left(36^{\circ },40^{\circ }\right)$, and $\left(39^{\circ },100^{\circ }\right)$. The pseudo-spectra in Figure 9 indicate that CSM and NUSM produce blurred ridges with uncertain localization. The Proposed LCCS recognizes certain source regions but generates duplicated and displaced peaks, indicating diminished stability with restricted snapshot support. The RASA accurately identifies all four genuine directions with distinct and separate peaks, validating its efficacy for direction-of-arrival estimate in arbitrary two-dimensional non-uniform arrays.

These tests illustrate that CSM and NUSM serve solely as baseline references and exhibit a lack of robustness when utilized in non-uniform geometries. The Proposed LCCS provides limited enhancement but is still contingent on geometry, faltering in NULA setups and producing unreliable estimates on NURA with a lower number of snapshots. Conversely, the RASA consistently attains precise and stable localization in both one-dimensional and two-dimensional non-uniform arrays, positioning it as the most dependable method for practical direction-of-arrival estimation.

### 6.3. Resolution Comparison: Numerical Example

To test the resolution capability of these four algorithms, a URA with 16 array elements (M=16) is used to detect DoAs of seven signal sources (L=7) of elevation angles (θ) given by $\theta =[-60^{\circ },-50^{\circ },-40^{\circ },0^{\circ },40^{\circ },50^{\circ },60^{\circ }]$ and the same azimuth for all sources, $\phi =0^{\circ }$.

**Figure 10:**
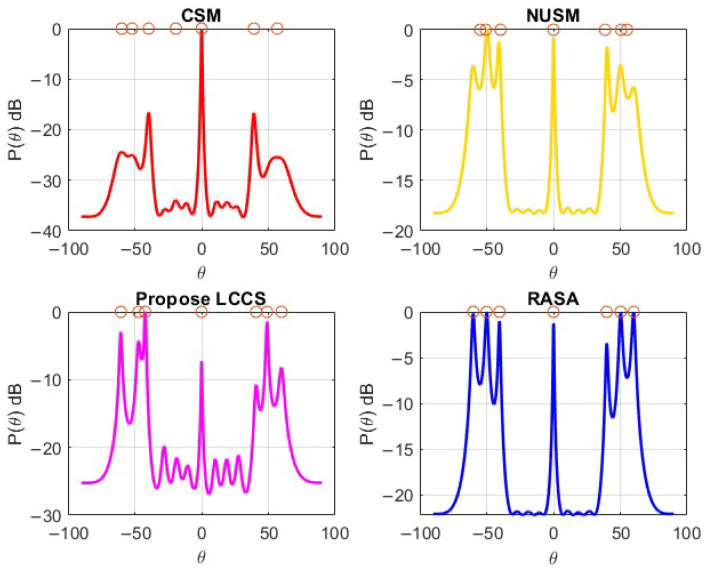
Resolution performance comparison of the RASA method with CSM, NUSM, and Proposed LCCS methodologies with seven DoAs. The two sets of three closely separated DoAs represent a severe test.

Figure 10 shows the detection performance of the algorithms for this specific case. P(θ) is the power of the signal at that specific angle θ. It is clear that the three negative and positive elevation-angled sources are closely located. A closer look reveals significant variations in resolution quality and accuracy, even if it could seem that all approaches identify the seven peaks in Figure 10. In particular, only RASA correctly detects the two clusters of closely spaced sources as well as all seven real DoAs. On the other hand, under-detection results from peak merging in those clusters for CSM and NUSM. The proposed LCCS shows six peaks, but it does not differentiate all closely spaced sources, and one of them is probably a false detection because of spectral noise. Because RASA was the only method among those tested that successfully resolved all sources with high accuracy and no observed false positives, the visual presence of seven peaks alone does not guarantee accurate detection.

As a result, only the RASA algorithm successfully resolves the two clusters of closely spaced sources, resulting in seven distinct and well-separated peaks without merging or false detection, even though all algorithms display numerous peaks. The other methods, on the other hand, show overlapping or wider peaks, which causes uncertainty in source localization. This illustrates RASA’s improved robustness and higher resolution capacity in situations with high-density sources.

### 6.4. Comparison Based on Different SNR Levels

This section examines how SNR affects the estimators’ performance. The SNR varies from −10 to 10 dB in 5 dB steps. In order to receive signals from L = 10 sources that are positioned at random throughout the angular sector of $\left[-90^{\circ },90^{\circ }\right]$, a ULA with M=32 sensors is used. To guarantee a consistent and equitable comparison, a total of K=500 Monte Carlo trials is carried out for each SNR level. In each trial, a fresh set of ten random DoAs is created and used uniformly across all methods. N=100 is the fixed number of data samples in each trial, and all estimators use the sample covariance matrix.

**Figure 11:**
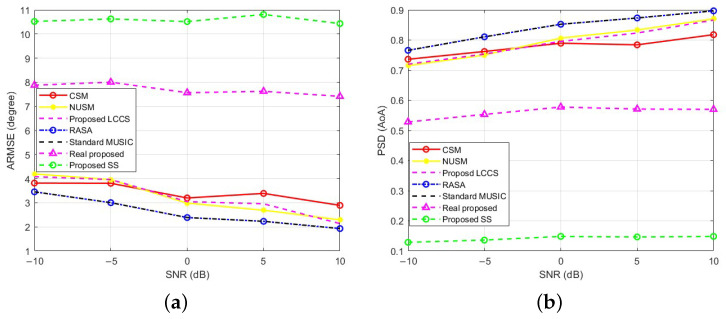
Performance comparison of DoA ARMSE (**a**) and PSD (**b**) vs. SNR.

An initial summary of the performance of all tested algorithms, including RASA, Standard MUSIC, Proposed LCCS, NUSM, CSM, Real Proposed, and Proposed SS, is shown in Figure 11a. RASA and Standard MUSIC have nearly identical ARMSE at all SNRs; the curves overlap completely, rendering the RASA trace visually indistinguishable. These two methods consistently achieve the lowest angular-estimation error. Similarly, Figure 11b shows that both methods attain the highest PSD and maintain high resolution across the entire SNR range. By contrast, Proposed SS and Real Proposed perform substantially worse, while Proposed LCCS, NUSM, and CSM are intermediate.

Similar to MUSIC, RASA is predicated on a recognizable noise subspace. With our setup (M=32, N=100, L=10), SNR significantly below −10dB results in the traditional threshold effect: Eigen-separation collapses, and the degradation of all subspace DoA estimators is comparable. Consequently, Figure 11 starts at −10dB, the lowest SNR at which the subspace is still usable in this configuration. In accordance with the reduced subspace column correlations shown in Table 1, RASA’s selection of the least-correlated noise-subspace columns results in a better-conditioned projector when the subspace is recognizable.

**Figure 12:**
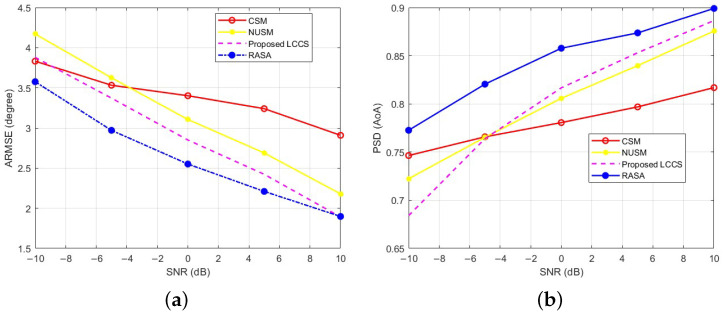
Performance comparison of DoA ARMSE (**a**) and PSD (**b**) vs. SNR for CSM, NUSM, Proposed LCCS, and RASA.

These conclusions lead us to exclude the lower-performing methods (Proposed SS and Real Proposed) from further analysis and to focus on the strongest baselines for a clearer comparison. Figure 12a compares RASA, Proposed LCCS, NUSM, and CSM; Standard MUSIC is omitted because its performance is indistinguishable from RASA in Figure 11. While RASA achieves the lowest ARMSE among the studied SNRs, Proposed LCCS comes in second, while NUSM and CSM show larger estimate errors, especially at low SNR. Likewise, Figure 12b demonstrates that in this configuration, RASA attains the maximum PSD throughout the sweep; Proposed LCCS comes in second, followed by NUSM and CSM. Everything is true within the operating SNR range, which in our arrangement is ≥−10 dB, where the noise subspace is discernible.

The results show RASA performing best on ARMSE and PSD in this setup; the following sections therefore concentrate on the strongest baselines.

### 6.5. Comparison Based on Different Numbers of Snapshots

**Figure 13:**
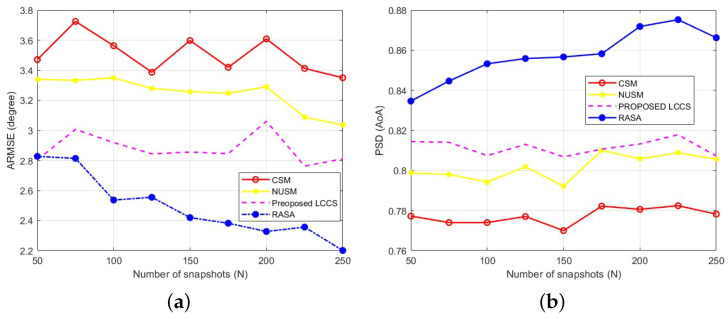
Performance comparison of the RASA algorithm with other sampling methodologies based on a different number of snapshots: ARMSE (**a**) and PSD (**b**).

The capability of direction-finding techniques to accurately determine the location of sources with a limited number of snapshots is crucial, as obtaining a large number of snapshots is not always feasible in many real-world applications. In this context, we investigate the impact of varying the number of snapshots on the estimation accuracy of the proposed sampling method, alongside its competing techniques. To do this, we set the SNR to 0 dB and construct the CM using different snapshot values: N=[50,75,100,125,150,175,200,225,250]. The number of sources and the receiver array properties remain the same as in the scenario presented in Section 6.3 (L=10, M=32). To ensure unbiased results, one thousand Monte Carlo trials are performed. For each value of *N*, the ARMSE and PSDs are computed, and the results are presented in Figure 13a and Figure 13b, respectively.

The detection capacity of the other algorithms (CSM, NUSM, and the suggested LCCS) stays almost constant as the number of snapshots grows, as Figure 13 makes evident. On the other hand, the RASA sampling technique consistently beats all benchmarks, obtaining higher detection probability and lower estimation error over the whole range of *N*. This demonstrates how RASA can use the correlation structure of the noise subspace columns to choose the most informative columns, resulting in a decorrelated and well-conditioned sampling matrix with a sharper MUSIC spectrum. Furthermore, it should be mentioned that the sub-degree ARMSE and PSD values that approach unity under more moderate settings are achieved by these methods, while the absolute levels (ARMSE $>1^{\circ }$, PSD <1) reflect the purposefully strict simulation setup (M=32, L=10, short snapshots). The slight oscillations observed in Figure 13a arise from the random generation of DoAs in each Monte Carlo trial and the finite number of realizations, which introduce small statistical variations even after averaging.

### 6.6. Comparison Based on Correlated Signals

Correlation between arriving signals leads to an increase in the Sidelobe Level (SLL) in array responses, which negatively affects direction-finding systems. To mitigate this effect, pre-processing techniques such as spatial smoothing are commonly employed. However, this solution introduces higher computational complexity. To maintain low computational costs, it is crucial that the technique can effectively handle correlated sources. This scenario examines the impact of signal correlations on the performance of the evaluated DoA techniques. For this, we assume that the incident signals are correlated due to multipath effects, with ten correlated signals (L=10) arriving at the same array as in the previous Section 6.3 and Section 6.4. Specifically, we assume that one signal is from the primary source, while the other signals are reflections of the first. To model this scenario, we vary the correlation level between the first and other signals using the following set of correlation coefficient values, CC=[0.1,0.2,0.4,0.6,0.8]. The SNR and the number of data samples are set to 4 dB and 100, respectively.

**Figure 14:**
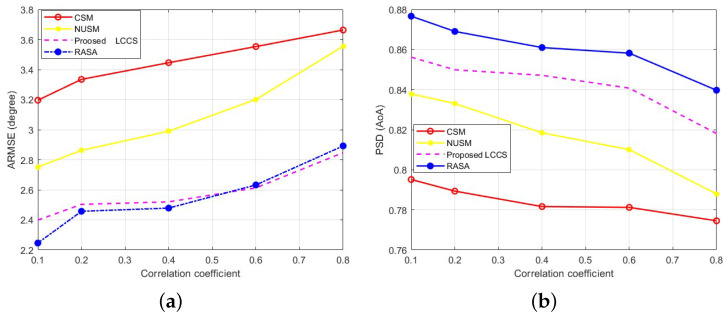
(**a**) Accuracy (ARMSE) comparison of the RASA algorithm with the other sampling methodologies based on different correlation levels between arrived signals; (**b**) illustration of the corresponding PSD (DoA).

Next, two thousand sets (K=2000) of ten DoAs are generated, with each set applied equally across all considered algorithms. For each trial, we compute ARMSE and PSD, then plot the results against the correlation coefficients CC, as shown in Figure 14a and Figure 14b, respectively. It is observed that the RASA algorithm achieves the highest accuracy compared to the other algorithms when the incident signals are either slightly (CC=0.1) or highly (CC=0.8) correlated. This indicates that the proposed sampling approach makes the PM method less sensitive and more robust to correlated signals by eliminating dependency on the steering vector. Furthermore, it is shown that the classical sampling method is the least robust to such signals, followed by the NUSM and Proposed LCCS methods, respectively.

### 6.7. Comparison Based on Different Array Aperture

The purpose of this scenario is to examine how different values of *M* influence the detection capability of the algorithms. Without loss of generality, the total number of equally spaced array elements is varied as: M=[15,20,25,35,40,50,75]. A simulation is conducted with the following parameters: L=10, SNR=0 dB, N=100, and K=500. Therefore, the total number of generated DoAs is 5000. These DoAs are applied equally across all the algorithms. The Percentage of Detected Angles (PDA) is calculated for each sampling method using the following formula as defined in Equation (44).

(44) $$\mathrm{PDA}\left(\%\right)=\frac{\mathrm{number}\;\mathrm{of}\;\mathrm{correctly}\;\mathrm{detected}\;\mathrm{angles}}{\mathrm{total}\;\mathrm{number}\;\mathrm{of}\;\mathrm{generated}\;\mathrm{angles}}\times 100$$

**Figure 15:**
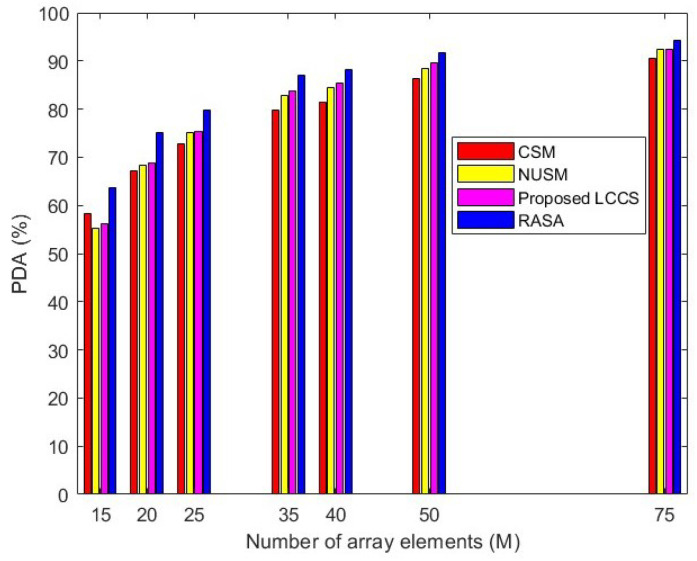
Performance comparison based on different array sizes.

The resulting data is presented in Figure 15. From the bar plot, it can be observed that a larger array size results in an increase in the aperture of the array, which in turn increases the number of detectable angles for all algorithms. The classical method exhibits comparable performance to the proposed method for a lower number of array elements. The NUSM and LCCS methods show moderate performance, with LCCS detecting more angles than the NUSM method. Notably, the RASA algorithm detects the highest number of angles, and this superior performance remains consistent for array sizes as small as 15 and as large as 75. This indicates that the proposed method outperforms the previous sampling techniques, regardless of whether the physical array size is small or large.

### 6.8. Comparative Evaluation: Standard MUSIC, RASA, and ML Post-Processors

A uniform linear array (ULA) including M=32 sensors and K=7 sources is examined, featuring inter-element spacing of λ/2 and N=100 snapshots. The MUSIC pseudo-spectrum is examined throughout the interval $\left[-90^{\circ },90^{\circ }\right]$ utilizing a grid of $1^{\circ }$ (181 bins). Uniform diagonal loading is implemented on the covariance matrix $R_{xx}$ across all methodologies. Test signal-to-noise ratios (SNRs) are {−10,−5,0,10} dB, assessed under both uncorrelated and coherent sources, with and without perturbation of element positions. The selected metrics are root-mean-square error (RMSE, degrees), probability of successful detection within $1^{\circ }$ (PSD), and average runtime per invocation. Three post-processors at the spectrum level function on the unchanged normalized MUSIC pseudo-spectrum: (i) a spectrum-to-sorted DoA vector mapping using an MLP regressor; (ii) an MLP multi-label classifier over S=24 angular sectors that yields the top-*K* sectors’ centers; and (iii) a streamlined 2D-CNN regressor with two fully connected layers, global average pooling, and [5×1] convolutions along the angle axis. All the details about these three algorithms can be found in [38,39,40]. To encourage generalization, models are trained on 4000 synthetic samples with random coherence/perturbation and SNR augmentation {−10,−5,0,5,10,15,20} dB. Each ML method’s inference cost is expressed as MUSIC time + model time; neither hybrid nor restricted scanning is employed.

**Figure 16:**
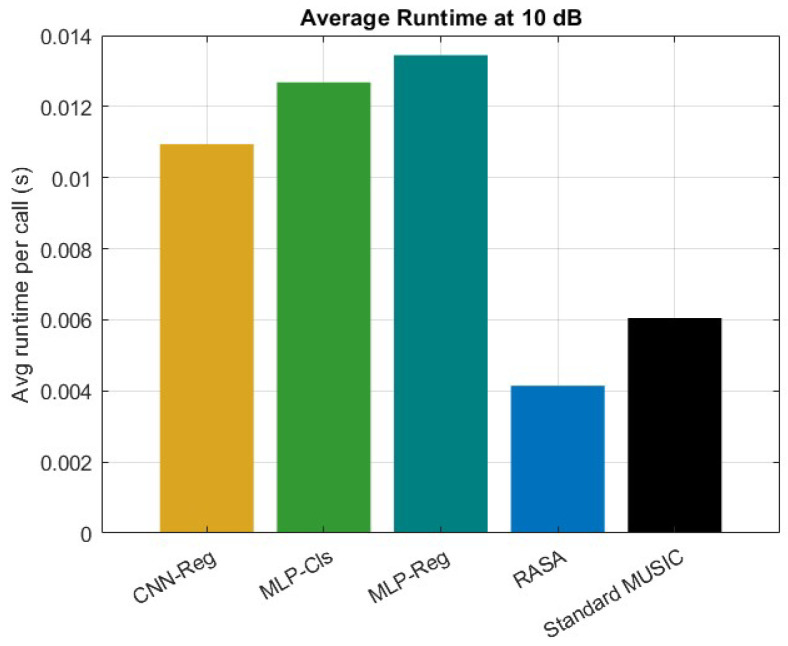
Average per-call runtime at 10 dB (ULA, M=32, K=7, N=100). Methods: Standard MUSIC, RASA, MUSIC+MLP (Reg), MUSIC+MLP (Cls), MUSIC+CNN (2D Reg).

The suggested method has a number of clear advantages over current ML-based DoA estimate methods. Initially, it circumvents the necessity of extensive labeled training datasets, which are generally necessary for neural network-based estimators like attention-driven models, convolutional neural networks (CNNs), and multilayer perceptrons (MLPs). As a result, the technique is less susceptible to domain mismatch and more data-efficient when used with various array topologies or propagation conditions. Second, unlike deep learning models, which frequently function as black boxes, the suggested approach preserves interpretability because the underlying pseudo-spectrum is still generated from subspace principles. Third, as shown by the runtime comparison in Figure 16, our method shows lower computational cost during inference, whereas ML-based methods require additional matrix pre-processing and neural computations.

**Figure 17:**
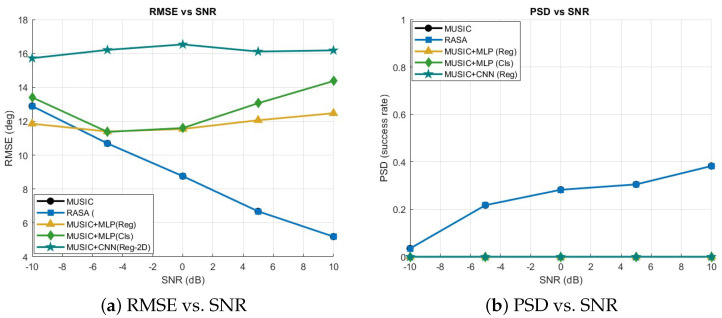
Comparison of (**a**) RMSE vs. SNR (ULA, M=32, K=7, N=100, scan $\pm 90^{\circ }$ at $1^{\circ }$) and (**b**) PSD vs. SNR (success if $\left|\Delta \theta \right|\leq 1^{\circ }$) for Standard MUSIC, RASA, and MUSIC+ML methods (MLP-Reg, MLP-Cls, CNN-Reg).

However, it is important to recognize some restrictions. Under very low SNR or highly coherent conditions, pure machine learning techniques can occasionally provide better robustness by learning to denoise or regularize the pseudo-spectrum. Moreover, without requiring spectrum scanning, ML models can produce extremely quick DoA estimations once they are trained. In order to address these issues, we provided comparison simulations in which the suggested approach was tested with MUSIC+ML hybrids (MLP-Reg, MLP-Cls, and CNN-Reg). The findings (Figure 16 and Figure 17) demonstrate that although machine learning techniques can marginally lower RMSE in some low-SNR situations, their effectiveness varies in the absence of substantial training data. On the other hand, the suggested approach continuously maintains a good balance between resolution, complexity, and generalization while achieving stable accuracy throughout SNR ranges.

The suggested RASA framework has the following advantages over ML-based DOA methods: it doesn’t require any training data, it ensures interpretability through eigen-structure analysis, and it maintains predictable complexity that scales solely with *M* and *N*. However, in extremely low-SNR or highly coherent situations, ML post-processors (MLP, CNN) can be useful and can generate continuous DOA estimations without grid scanning. The variations in RMSE, PSD, and runtime findings shown in Figure 16 and Figure 17 can be explained by these trade-offs.

## 7. Conclusions

In this study, a new DoA estimation algorithm, denoted as RASA, is proposed, and its performance was thoroughly evaluated through extensive simulation results. The simulations demonstrated the method’s consistently better performance across the tested scenarios, including 2D DoA estimation, resolution comparison, varying SNR levels, snapshot counts, correlated signals, and different array apertures. The proposed method, based on MUSIC with QLCCS, consistently outperformed other traditional techniques such as CSM, NUSM, and Proposed LCCS in terms of both accuracy and robustness.

In particular, the proposed method excelled in resolving closely spaced DoAs, as demonstrated by its ability to detect up to seven sources with high precision, even when the sources were in close proximity. In comparison, the other methods struggled to resolve multiple closely spaced sources, leading to missed detections or ambiguity in the DoA estimates. Moreover, the proposed method exhibited significantly better performance in terms of Average Root Mean Square Error (ARMSE) and Probability of Successful Detection (PSD) when subjected to varying SNR levels, demonstrating its robustness in noisy environments.

The results also highlighted the method’s resilience in situations involving correlated signals, where it outperformed other algorithms by maintaining high estimation accuracy despite increasing correlation coefficients. Furthermore, the proposed technique demonstrated remarkable performance even with a limited number of snapshots, proving its practical applicability in real-world scenarios where data acquisition may be constrained.

Overall, the proposed DoA estimation method proves to be a reliable and efficient solution for a wide range of practical applications, including communications and radar systems, where accurate direction-finding and source localization are crucial. The method’s ability to handle various real-world challenges, such as signal correlations, limited snapshots, and closely spaced sources, sets it apart from existing approaches, making it a promising tool for future advancements in DoA estimation. In summary, the RASA approach has demonstrated consistently better performance in terms of estimation accuracy and detection probability across a wide range of simulated scenarios. The next stage of this research will employ real passive radar data, with particular emphasis on challenging coherent-source conditions. This experimental validation will allow us to confirm the robustness of the method under practical factors such as hardware impairments, array calibration, and environmental uncertainties. In parallel, hybrid strategies that integrate ML-based denoising with the proposed subspace framework will also be explored, aiming to further improve robustness in low-SNR and highly coherent scenarios.

## Figures and Tables

**Table 1:** Mean absolute Pearson correlation $|\rho_{q_{i},q_{i+1}}|$ between adjacent selected columns $\left(q_{i},q_{i+1}\right)$ using different subspace sampling techniques.

Classical ($Q_{\mathrm{CSM}}$)		Uniform ($Q_{\mathrm{USM}}$)		Non-Uniform ($Q_{\mathrm{NUSM}}$)		Proposed LCCS ($Q_{\mathrm{LCCS}}$)	
$\left(q_{i},q_{i+1}\right)$	$|\rho_{q_{i},q_{i+1}}|$	$\left(q_{i},q_{i+1}\right)$	$|\rho_{q_{i},q_{i+1}}|$	$\left(q_{i},q_{i+1}\right)$	$|\rho_{q_{i},q_{i+1}}|$	$\left(q_{i},q_{i+1}\right)$	$|\rho_{q_{i},q_{i+1}}|$
1, 2	0.1506	1, 2	0.1503	1, 2	0.1496	1, 2	0.1500
2, 3	0.1502	2, 3	0.1509	2, 3	0.1445	2, 3	0.1517
3, 4	0.1507	3, 4	0.1539	3, 4	0.1453	3, 4	0.1522
4, 5	0.1485	4, 5	0.1498	4, 5	0.1545	4, 5	0.1511
5, 6	0.1519	5, 6	0.1448	5, 6	0.1519	5, 6	0.1523
6, 7	0.1425	6, 7	0.1502	6, 7	0.1491	6, 7	0.1494
7, 8	0.1429	7, 8	0.1498	7, 8	0.1456	7, 8	0.1459
8, 9	0.1498	8, 9	0.1530	8, 9	0.1486	8, 9	0.1483
9, 10	0.1503	9, 10	0.1466	9, 10	0.1499	9, 10	0.1478
**RASA** ($Q_{\mathrm{RASA}}$)		**Proposed SS** ($Q_{\mathrm{SS}}$)		**Real Proposed** ($Q_{\mathrm{Real}}$)			
$\left(q_{i},q_{i+1}\right)$	$|\rho_{q_{i},q_{i+1}}|$	$\left(q_{i},q_{i+1}\right)$	$|\rho_{q_{i},q_{i+1}}|$	$\left(q_{i},q_{i+1}\right)$	$|\rho_{q_{i},q_{i+1}}|$		
1, 2	0.0705	1, 2	0.0896	1, 2	0.1018		
2, 3	0.0675	2, 3	0.1042	2, 3	0.1064		
3, 4	0.0709	3, 4	0.0933	3, 4	0.1059		
4, 5	0.0800	4, 5	0.1076	4, 5	0.1077		
5, 6	0.0822	5, 6	0.1100	5, 6	0.1073		
6, 7	0.0867	6, 7	0.1071	6, 7	0.1027		
7, 8	0.0955	7, 8	0.1063	7, 8	0.1058		
8, 9	0.0927	8, 9	0.1085	8, 9	0.1025		
9, 10	0.0958	9, 10	0.1103	9, 10	0.1028		

**Table 2:** Wilcoxon signed-rank test results comparing RASA against baseline methods. Reported values include median difference (DeltaMedian), *p*-value, and effect size (*r*).

Baseline	DeltaMedian	*p*	Effect *r*
CSM	−0.0553	$1.77\times 10^{-164}$	0.864
USM	−0.0569	$4.08\times 10^{-164}$	0.863
NUSM	−0.0570	$8.88\times 10^{-163}$	0.860
LCCS	+0.0106	$2.62\times 10^{-95}$	0.655

**Table 3:** Real-valued vomputational FLOPs and algorithm complexity.

Algorithm	FLOP Expression (Real Domain)	Subspace Size	Complexity
Standard MUSIC	$4M^{2}\left(L+2\right)+4J\left(M+1\right)\left(M-L\right)$	M−L	Moderate
Real Proposed	$M^{2}\left(2L-K+2\right)+J\left(M+1\right)\left(M-2L+K\right)$	M−2L+K	Reduced
Proposed LCCS	$M^{2}+ML+L^{3}+J\left(M+1\right)$	L+2	Low-Medium
Proposed SS	$M^{2}N+M^{3}+P^{2}S^{2}+P^{3}+JP^{2}$	P−L	High
RASA	$M^{3}+M^{2}+ML+J\left(M+1\right)$	L+2	Low

## Data Availability

No new data were created or analyzed in this study.
